# Precision Nutrition for Public Health

**DOI:** 10.3390/nu17183004

**Published:** 2025-09-19

**Authors:** Sabina Lachowicz-Wiśniewska, Agata Kotowska

**Affiliations:** 1 Department of Medical and Health Science, University of Kalisz, W. Bogusławskiego 2 Square, 62-800 Kalisz, Poland; 2 Department of Biotechnology and Food Analysis, Wroclaw University of Economics and Business, Komandorska 118/120 Street, 53-345 Wroclaw, Poland; 3 Institute of Sociology, University of Rzeszów, Rejtana 16C, 35-959 Rzeszów, Poland

## 1. Introduction

Public health—understood as both a science and a practice aimed at preventing disease, prolonging life, and promoting health—has become one of the most critical domains of institutional action, shaped by both nation-states and international organizations. These activities include, in particular, population health assessment, epidemiological surveillance, health promotion, disease prevention (environmental and infectious), and emergency preparedness. Conducting interdisciplinary research across these domains underpins key public health decisions—both ad hoc and those concerning the formulation of long-term policies designed to protect and support population health. In view of rapid societal change and the emergence and consolidation of phenomena that adversely affect human health, undertaking in-depth scientific studies and implementing the resulting recommendations are especially warranted.

In this special issue of *Nutrients* entitled “Public Health, Dietary Behaviors, and Nutritional Status,” we present findings generated by scholars from multiple disciplines using diverse research methodologies. Some contributions are reviews, while others report original studies (pilot, representative, and non-representative). The papers identify selected determinants of population health and correlations among variables, addressing health-related social behaviors including established habits, knowledge, self-perception, and the societal perception of individuals with obesity.

## 2. Results

Considerable attention is devoted to obesity, a highly prevalent disease in contemporary societies associated with numerous serious health consequences. The contributors argue that conventional indices (body mass index and waist circumference) are insufficient for characterizing early metabolic risk in patients with obesity, and that the development of new, more precise indicators is warranted. Accordingly, the studies focus on the impact of abdominal adipose tissue (subcutaneous and visceral) on disturbances of carbohydrate and lipid metabolism in individuals with obesity. This impact proved to be significant; however, ambiguous findings regarding dyslipidemia warrant further research in larger populations [contribution 1]. At the same time, mHealth practices—by intervening in users’ lifestyles—may facilitate health promotion and reduce the risk of cardiometabolic syndrome. Study participants demonstrated improvements in health behaviors, with improved healthy-eating scores and increased physical activity indices. Health risk factors declined substantially, and the proportion of individuals with three or more components of the metabolic syndrome was halved (from 42.3% at baseline to 19.2% at endline) [contribution 2].

Obesity, in addition to its adverse effects on physical health, also affects mental health and self-esteem. Cultural beauty standards often subject individuals living with obesity to social stigmatization, and bias and stigma may even be observed at times among physicians, dietitians, and other healthcare professionals [contribution 3]. Overweight and obesity markedly reduce the likelihood of satisfaction with one’s body and health, and among women, a history of depression and emotional eating increased the odds of overweight/obesity [contribution 4]. Body satisfaction is higher among physically active individuals, although its distribution is shaped differently in women and men. Studies in strength-training populations have revealed significant sex differences across multiple domains, including body satisfaction, dietary patterns, and psychological traits [contribution 5].

Lifestyle is among the most important determinants of health, with diet constituting a principal component and dietary modification therefore being a common strategy for improving quality of life, well-being, self-image, and social acceptance. The crucial roles of the gut microbiota, dietary habits, and physical activity have been demonstrated in the management of co-occurring depression and obesity [contribution 6]. In the media (particularly online), one frequently encounters vigorous promotion by proponents of various elimination diets; however, their use carries the risk of deficiencies in essential nutrients. Studies among healthy adults adhering to omnivorous, vegetarian, vegan, and low-carbohydrate–high-fat diets indicate that micronutrient deficiencies are common—regardless of dietary pattern—even though participants believe their diets are balanced and health-promoting. In such cases, appropriately selected dietary supplements may increase the likelihood of meeting micronutrient intake recommendations [contribution 7]. Research on diet and nutrient deficiencies in Brazilian adults shows that, despite high rates of adequacy for macronutrients (over 99% for protein, 84.7% for carbohydrates, and 80.7% for total fat), there remains a substantial prevalence of micronutrient inadequacy, to which women and older adults are particularly vulnerable. The population exhibited a high probability of inadequacy for vitamins D and E (above 90%) and for calcium and magnesium (above 85%), irrespective of age group. Except for iron, the probability of inadequacy for other minerals increased with age [contribution 8]. Similarly, in the United States population, inadequate calcium intake is widespread; moreover, dietary calcium intake declined between 2009 and 2018 [contribution 9].

One dietary approach gaining worldwide popularity is intermittent fasting, which has favorable effects on metabolic health and weight reduction. In a Spanish cohort, individuals practicing intermittent fasting reported better body image and exhibited greater discipline with respect to lifestyle and diet. Most participants were middle-aged men, and no significant differences were observed by educational attainment or place of residence [contribution 10]. In Spain, plant-based eating is also becoming increasingly common, gradually supplanting the Mediterranean dietary pattern, and studies indicate that sociodemographic variables do not influence its prevalence. Compared with adherents of the Mediterranean diet, individuals following vegetarian or vegan diets have lower BMI, consume less processed food, and lead healthier lifestyles (sleep longer, exercise more, smoke less, and consume alcohol less frequently); however, they display a higher prevalence of diagnosed eating disorders [contribution 11].

Given adverse public health trends in Lebanon—rising rates of obesity, undernutrition, and other noncommunicable diseases among adults—an assessment of dietary habits was undertaken. The findings indicate that the Lebanese diet is characterized by high energy intake, elevated consumption of sugars, sodium, and saturated fats, and insufficient intake of healthy fats and vitamins A, D, and E. Most respondents were overweight or obese, and adult women failed to meet daily intake requirements for calcium, vitamin D, iron, and vitamin B12, increasing the risk of anemia, osteoporosis, and other health problems [contribution 12].

Excessive alcohol consumption—linked to elevated risks of cancer, mental disorders, and aggression—represents a particularly detrimental population health phenomenon. In Brazil, alcohol use is hazardous and exceeds the global average by nearly 30%. A study comparing the costs of consuming the most popular alcoholic beverages with expenditures on basic food staples showed unequivocally that all modeled scenarios of high alcohol consumption cost less—or far less—than a basic 2000 kcal/day diet. This analysis underscores the urgent need for fiscal policy establishing a minimum unit price for alcohol to reduce its affordability [contribution 13].

Access to safe drinking water is an increasingly important global health issue. A study of Italians’ consumption of tap water—premised on the notion that the production and consumption of bottled water contribute to water scarcity and pollution—evaluated attitudes toward drinking tap water in relation to sociodemographic factors, obesity, and self-perceived health status [contribution 14].

Because a large share of daily life occurs at work or school, there is a clear need to shape and improve dietary habits within occupational and educational settings [contribution 15]. For example, a study of Portuguese university students found comparable levels of two behaviors: regular use of vending machines and bringing lunches from home (70% and 60%, respectively) [contribution 16].

Despite educational programs, public campaigns, and relatively easy access to information, knowledge about how nutrition affects human functioning and health should be continuously disseminated, as in many cases understanding may depend substantially on sociodemographic variables. Nutritional knowledge is also highly useful for improving quality of life and alleviating certain conditions—for example, dietary patterns can mitigate or exacerbate menstrual symptoms [contribution 17]. In the context of women’s health, research has also examined the association between cervical cancer and dietary habits [contribution 18].

Possessing nutrition knowledge is particularly important for individuals with specific dietary needs, such as adolescents engaged in sports. A study assessing sports nutrition knowledge among parents and guardians of male academy footballers in the United Kingdom demonstrated both the feasibility and the necessity of intervention in this area, with nutrition knowledge classified as “poor” overall [contribution 19]. Because athletes’ nutrition involves specialized and often costly requirements, meeting these needs becomes a critical issue for both health and athletic performance. Studies among student athletes in the United States indicate that food insecurity (FI) is a real and pressing problem: depending on the region, FI prevalence ranged from 9.9% to 65%, influenced by limited financial resources, time management, meal-plan structures, and housing location/amenities [contribution 20]. Public health challenges do not stem solely from excess or poor-quality food; they are equally tied to scarcity. An analysis of children under five in various agro-ecosystems of northwestern Ethiopia found an overall undernutrition prevalence of 49% in the study population, with marked variation by agro-ecosystem (from 36.1% in highland areas to 59% in lowland areas) [contribution 21].

In this context, pediatric data are particularly important in showing that excess body weight is not the only determinant of early cardiometabolic risk. A study of Polish children aged 5–11 years born small for gestational age (SGA; *n* = 93), compared with appropriate-for-gestational-age (AGA) peers (*n* = 47), found that, despite no differences in body weight or BMI, SGA children exhibited an adverse metabolic profile: higher total cholesterol, LDL cholesterol, and triglycerides, as well as higher fasting glucose. No differences in the lipid profile were observed between SGA subtypes (symmetric vs. asymmetric IUGR), and the only significant difference was higher fasting glucose in symmetric SGA. These findings strengthen the argument that, in public health practice, cardiometabolic markers should be routinely assessed in children with an SGA history—irrespective of their current anthropometric indices—aligning with the logic of early cardiovascular disease (CVD) prevention [contribution 22].

The broad spectrum of issues addressed in the contributions presented here not only illustrates how wide-ranging the field of public health is, but it may also provide a foundation for formulating long-term policies and for taking concrete practical actions at local and global levels.

## 3. Conclusions

This Special Issue synthesizes current public health themes at the intersection of nutrition, lifestyle, and the prevention of chronic diseases. The authors emphasize that conventional indices (BMI, waist circumference) are insufficient for early cardiometabolic risk assessment and call for more precise markers, including the evaluation of fat distribution (subcutaneous and visceral). They highlight the effectiveness of mHealth interventions in modifying health behaviors and reducing metabolic syndrome components, as well as the psychosocial consequences of obesity (stigma, effects on self-esteem), with sex differences in body satisfaction and habits. The studies presented demonstrate the widespread nature of micronutrient deficiencies across diverse dietary patterns (from omnivorous to vegan and low-carbohydrate–high-fat), the growing interest in intermittent fasting and plant-based diets, and the national and environmental determinants of diet (Lebanon, Brazil, Ethiopia, Italy). In the area of nutrition education, the contributions underscore knowledge gaps (e.g., among parents of youth athletes), food insecurity among student athletes, and the importance of diet in menstrual symptoms and cancer prevention. A particularly important pediatric point is that children born SGA exhibit an unfavorable metabolic profile despite normal BMI, which justifies early cardiometabolic screening in this group. Taken together, the evidence supports multilevel, systemic actions—from fiscal and environmental policies to personalized nutrition and education strategies.

The key theses and practical implications are as follows:

- Beyond BMI: There is a need for markers that more precisely reflect risk (visceral adiposity, metabolic indicators).
- mHealth works: Digital interventions effectively improve diet and physical activity, reducing components of MetS.
- Psychosocial dimension: Obesity stigma (including within healthcare) undermines well-being, and anti-bias measures are needed.
- Diets and deficiencies: Micronutrient shortfalls persist across many dietary patterns—supporting targeted supplementation and education.
- National context: Food and fiscal policies (e.g., minimum unit pricing of alcohol), improving population dietary habits, and access to healthy food/safe water should be public health priorities.
- Pediatrics (SGA): Routine lipid–glucose screening is indicated in children with an SGA history, regardless of BMI.

Overarching conclusion: This Special Issue outlines a “public health 2.0” agenda—integrating population-level policies with precision prevention, and treating biology (markers), behaviors (diet/physical activity), psychology (stigma), and environment (availability of healthy choices) as co-equal pillars.

